# Long-Term Effect of Having a Child at Risk of Developmental Delays on Parental Labor Force Participation

**DOI:** 10.1007/s10995-024-03897-4

**Published:** 2024-02-09

**Authors:** Yanan Hu, Emily Callander

**Affiliations:** 1 Monash Centre for Health Research and Implementation, School of Public Health and Preventive Medicine, Monash University, Melbourne, VIC Australia; 2 University of Technology Sydney, Building 10, Level 8, Room 109 235-253 Jones Street, Ultimo, NSW 2007 Australia

**Keywords:** Children, Developmental delay, Labor force participation

## Abstract

**Objective:**

This study aimed to examine the long-term influence of having a child at risk of different developmental delays (communication, mobility, self-care, relating, learning, coping, or behaving) on parental labor force participation as the child grows.

**Method:**

A retrospective cohort was conducted using data from the Longitudinal Study of Australian Children survey, Waves 1–8 covering birth to 15 years of age of children. Multivariable logistic regressions were used to explore the odds ratio of mothers being out of the labor force at different children’s ages. Cox proportional hazards models were utilized to identify the ‘risk’ of mothers *returning* to the workforce after leaving. All models were adjusted for the mother’s age, education attainment, and employment status at time of birth, as well as marital status at the current wave.

**Results:**

There were 5,107 records of children, and 266 of them were at risk of any developmental delays at age 4–5 years. This sample represents 243, 026 children born in Australia in 2003/04. After adjusting for potential confounders, mothers of children at risk of each type of developmental delay (except mobility and self-care) had greater odds of being out of, and not *returning* to the labor force from children aged 2–3 to 14–15 years, when compared to mothers of children who are not at risk of developmental delays. Similar differences were found for fathers but were distinctly small and with narrower fluctuations, compared to mothers.

**Conclusion:**

Policies and programs funded by the government are greatly needed to support the mothers of children at risk of developmental delays.

**Supplementary Information:**

The online version contains supplementary material available at 10.1007/s10995-024-03897-4.

## Significance

**What is already known:**

Maternal labour force participation has been negatively affected by having a child with chronic health conditions, whereas paternal labour force participation is not significantly influenced.

**What this paper adds:**

Having a child at risk of developmental delays negatively influenced maternal and paternal labour force participation, where the influence on fathers was distinctly small and with narrower fluctuations during the 14 to 15 years of follow-up.

**Supplementary Information:**

The online version contains supplementary material available at 10.1007/s10995-024-03897-4.

## Introduction

There are wide variations in children’s achievement of key developmental milestones (Choo et al., 2019; World Health Organization, 2007, 2012). Developmental delays mean children continually lag in acquiring skills compared with peers at the same age, and this affects their ability to perform daily routines and activities (Choo et al., 2019; Fernald et al., 2009; World Health Organization, 2007). Although the condition itself may not be permanent, some significant developmental delays are signs of, or associated with a higher risk of, subsequent life-long conditions, such as autism, cerebral palsy, Down syndrome, and intellectual disability (Baker et al., 2002; Choo et al., 2019; World Health Organization, 2007). Therefore, early childhood development is closely related to a child’s later health and wellbeing (Lu et al., 2016). Both developmental delays and chronic health conditions increase health service utilization (e.g., hospitalization, and visits to physicians) (Arim et al., 2017; Gallaher et al., 2002; Russell et al., 2020).

However, focusing only on the direct costs of health service use takes a narrow view of the financial impact on families. Developmental delays and chronic health conditions also bring considerable challenges (e.g., physical and mental health issues of caregiver (Miodrag & Hodapp, 2010), impose indirect economic costs (e.g., opportunity costs arising from informal caregiving (Mitterer et al., 2021), and influence the employment status of parents – loss of income to families (Stabile & Allin, 2012; Zwicker et al., 2017).

Current evidence shows that parents’ labor force participation rate of those who have children with chronic health conditions was significantly lower than their counterparts (i.e., children without chronic health conditions) (Callander et al., 2019; Callander & Lindsay, 2018; Kish et al., 2018; Spiess & Dunkelberg, 2009), particularly among mothers – the main caregiver in most families of children with chronic health conditions (Toledano-Toledano & Luna, 2020). Previous findings also suggest that mothers face larger reductions in their involvement in the labor force when their children’s health problem is more severe, or they experience multiple health issues (Anderson et al., 2007; Burton et al., 2014; Powers, 2003). In addition, the age of children with chronic health conditions has been found to influence maternal labor force participation, with the mother’s labor force participation affected greatly for those children who were younger (Okumura et al., 2009). However, little is known about the impact of different types of children’s developmental delays on maternal and paternal labor force participation. Also, most previous research has focused on the mother’s labor force participation (Breslau et al., 1982; Corcnan et al., 2005; Kuhlthau et al., 2005; Kuhlthau & Perrin, 2001; Powers, 2003), overlooking the influence on the father’s workforce participation.

This study aimed to examine the influence of having a child at risk of different developmental delays on parental labor force participation. By considering how the labor force participation of both mothers and fathers is influenced by having children at risk of developmental delays, this study broadens the research focus of existing studies. This study also tracks the same individuals over a long follow-up time (birth to 15 years of age), which allows for a better understanding of the long-term effects on parental labor force participation.

## Methods

### Study Dataset

Data from the longitudinal survey, Growing Up in Australia: the Longitudinal Study of Australian Children (LSAC) (Australian Institute of Family Studies, 2015; Mohal et al., 2021), was utilized in this study. It is conducted by the Department of Social Services, the Australian Institute of Family Studies, and the Australian Bureau of Statistics. The LSAC survey collects information every two years (referred to as a ‘Wave’) on children’s health and development from parents, child carers (anyone who gives care and support to a relative or friend who has a medical condition (Department of Social Services, 2022), teachers, and the children themselves, plus information on mother’s and father’s labor force, education attainment and marital status, as well as other information not included in this study.

### Study Population

This study used information on the B-cohort (‘Baby’ cohort) between Waves 1–8, which contains data on 5,107 children from birth to 14–15 years. The LSAC survey, based on a complex probability sample, is specifically designed to produce valid estimates at the population level. The longitudinal weights for the sample that has responded to all waves of the survey were used to represent 243, 026 Australian population who were born in 2003/04. These weights take into account both the probability of selecting each child in the study and an adjustment for non-response.

### At Risk of Developmental Delays

From Wave 3 (children aged 4–5 years), parents completing the LSAC for the study child were asked a multiple-choice question: ‘Does the Study Child have a difficulty or delay in any of the following areas compared to children of a similar age?’, with the response options being:

- Communication (understanding or being understood by others);
- Mobility (getting out of bed, moving around home or at places away from home);
- Self-care (eating, drinking, dressing, bathing);
- Relating (interacting or playing with others);
- Learning (difficulty learning);
- Coping (coping with emotions);
- Behaving (managing his/her behavior); or.
- Other (everyday activities).

Based on the answers at Wave 3, we grouped children into ‘Not at risk of developmental delays’ or ‘At risk of any developmental delays’. We further separated the children ‘At risk of any developmental delays’ into ‘At risk of one developmental delay’, or ‘At risk of two developmental delays’, or ‘At risk of three or more developmental delays’ to assess the impact of multiple delays. To explore the effect of the specific type of delays, we also separated the children ‘At risk of any developmental delays’ into non-mutually exclusive seven subgroups based on the type of delays.

### Labor Force Status

From Wave 1 (children aged 0–1 years), the LSAC asked mothers and fathers of the subject child about their current labor force status, with the response options being ‘employed’, ‘unemployed’ and ‘not in the labor force’. The difference between ‘unemployed’ and ‘not in the labor force’ is that those who are ‘unemployed’ are actively seeking employment, whereas those who are ‘not in the labor force’ are not actively seeking employment (Australian Bureau of Statistics, 2007).

Our study focused on those who were ‘not in the labor force’ and grouped ‘employed’ and ‘unemployed’ as in the labor force.

### Statistical Analyses

We initially undertook a descriptive analysis of the study child and their parents to identify differences in demographic characteristics at time of birth. The proportion and odds ratio (estimated based on multivariable logistic regression models) of mothers being out of the labor force when the children were aged from 0 to 1 to 14–15 years were presented. We constructed a series of cox proportional hazards models showing the median length of time between leaving and returning to the labor force, and ‘risk’ of *returning* to the labor force after leaving. For mothers leaving and returning to the labor force more than one time during the follow-up, only the duration of the first time was considered. All models were adjusted for the mother’s age, education attainment, and employment status at time of birth, as well as marital status at the current wave. Fathers were not included in hazards and regression models due to the small sample size of fathers being not in the labor force.

All analyses were undertaken using SAS V9.4. Weighted results were reported unless otherwise stated. This study was reported following STROBE guidelines (Cuschieri, 2019). No research on human subjects was conducted, so ethics approval was not required. All research was conducted in accordance with the principles outlined in the Declaration of Helsinki. Patients’ consent to participate and for publication did not apply to our study.

## Results

In our sample, 266 (5.2%) children were at risk of any developmental delays at age 4–5 years, representing 16,621 children born in Australia in 2003/04. Of them, most children were at risk of *communication* delay, whereas the least were at risk of *mobility* delay. A similar number of children were at risk of single or ≥3 developmental delays Table 1.

**Table 1:** Sample and weighted number and percentage of children in B-cohort of the LSAC survey, in 2003/04, stratified by children’s health status at age 4–5 years

Health status at age 4–5 years	Sample number, n (%)	Weighted number, n (%)
Not at risk of developmental delays	4,841 (94.8)	226,405 (93.2)
At risk of *any* developmental delays	266 (5.2)	16,621 (6.8)
At risk of *one* developmental delay	111 (2.2)	6,774 (2.8)
At risk of *two* developmental delays	45 (0.9)	2,873 (1.2)
At risk of *three or more* developmental delays	110 (2.2)	6,975 (2.9)
At risk of *communication* delay	216 (4.2)	14,023 (5.8)
At risk of *mobility* delay	15 (0.3)	676 (0.3)
At risk of *self-care* delay	66 (1.3)	3,890 (1.6)
At risk of *relating* delay	92 (1.8)	5,068 (2.1)
At risk of *learning* delay	99 (1.9)	5,861 (2.4)
At risk of *coping* delay	92 (1.8)	4,739 (2.0)
At risk of *behaving* delay	90 (1.8)	6,087 (2.5)

Table 2 describes the demographic characteristics of children and their parents at time of birth. Compared to children who were not at risk of developmental delays at age 4–5 years, children of other health statuses were more likely to be male and their parents were younger.

**Table 2:** Children’s and their parents’ demographic characteristics at time of birth, stratified by children’s health status at age 4–5 years

Health status at age 4–5 years	Male, (%)	Aboriginal and/or Torres Strait Islander, (%)	No. of people in household, mean, SD	Mothers’ marital status married/de-facto, (%)	Mothers’ age, mean, SD (years)	Fathers’ age, mean, SD (years)	Mothers completed Year 12, (%)	Fathers completed Year 12, (%)
Not at risk of developmental delays	51.0	3.2	4.0 (10.7)	93.2	31.5 (49.2)	34.2 (54.0)	61.9	54.9
At risk of *any* developmental delays	66.4	5.7	4.1 (10.5)	93.1	30.3 (55.1)	33.3 (59.8)	49.5	44.6
At risk of *one* developmental delay	70.4	9.5	4.3 (10.7)	95.3	31.0 (47.1)	33.7 (59.3)	44.0	39.0
At risk of *two* developmental delays	63.3	10.4	4.2 (12.1)	89.4	29.6 (62.0)	33.4 (64.6)	36.5	32.8
At risk of *three or more* developmental delays	63.8	0	3.8 (9.0)	92.5	29.9 (60.6)	32.8 (59.4)	60.3	55.0
At risk of *communication* delay	64.9	4.6	4.1 (11.0)	92.1	29.9 (52.3)	32.9 (55.8)	49.3	42.3
At risk of *mobility* delay	71.8	0	3.5 (4.9)	100.0	27.6 (62.0)	27.9 (59.8)	69.1	73.4
At risk of *self-care* delay	58.2	7.7	3.7 (10.3)	100.0	29.2 (50.5)	32.0 (64.8)	60.6	54.9
At risk of *relating* delay	64.9	0	3.9 (9.6)	95.6	30.9 (51.0)	34.1 (57.1)	71.7	60.4
At risk of *learning* delay	56.5	0	4.1 (11.0)	94.7	30.1 (64.2)	34.1 (61.1)	55.8	47.7
At risk of *coping* delay	66.8	0	3.9 (9.3)	93.2	30.9 (47.1)	32.7 (46.3)	71.5	59.3
At risk of *behaving* delay	67.8	4.9	3.8 (9.4)	89.6	30.4 (61.7)	33.2 (58.1)	50.0	41.2

**Fig. 1:**
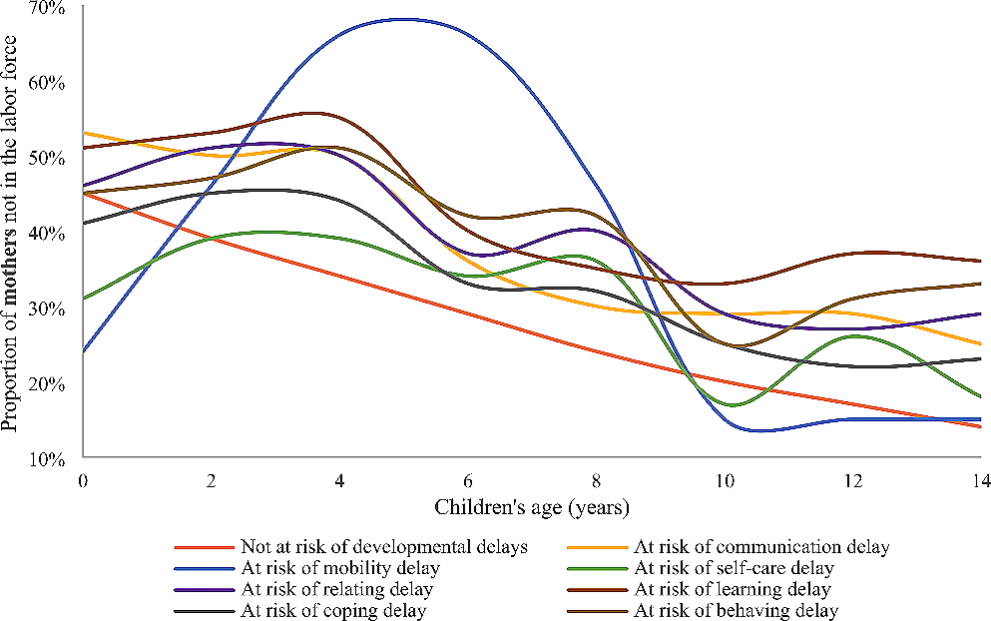
Proportion of *mothers* not in the labor force by risk of developmental delays at age 4–5 years and age of children

Figure 1 and S1 depict the proportion of mothers who were out of the labor force at different time points. These capture the labor force participation of mothers at that single point in time, so includes mothers who may have returned to the workforce, but then left again. Mothers of children at risk of each type of developmental delay consistently had a higher proportion of not being in the labor force than those children who were not at risk of developmental delays at each age across 2–3 to 8–9 years (Fig. 1). The highest proportions were observed for mothers of children at risk of ≥3 developmental delays at each age from 2 to 3 to 8–9 years (Figure S1).

Generally, there was a consistent decline in the proportion of mothers who were out of the workforce, as the children increased in age, regardless of health status. There were several fluctuations during the declining path. It was apparent that for children at risk of each type of developmental delay except for *mobility*, the proportion of mothers being out of the workforce slightly increased from children aged 0–1 years, peaked first at children aged 4–5 years, then dramatically declined, and peaked again at children aged 8–9 years, after that, dropped gradually with smaller fluctuations (Fig. 1). The proportion of being out of the labor force for mothers who had children at risk of *mobility* delay greatly fluctuated and reached as high as 66% at the children’s age of 4–5 and 6–7 years (Fig. 1).

**Fig. 2:**
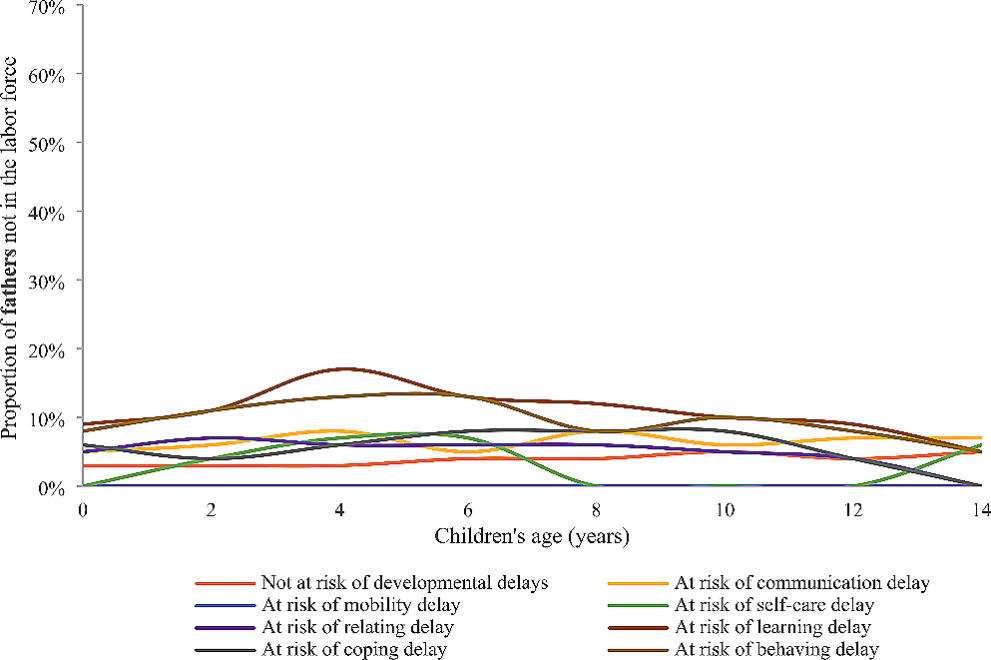
Proportion of *fathers* not in the labor force by risk of developmental delays at age 4–5 years and age of children

Compared to the proportion of mothers not in the labor force, the proportion of fathers being out of the labor force was distinctly small and had narrower fluctuations (Fig. 2 and S2). Fathers of children at risk of each type of developmental delay except mobility and self-care consistently had a higher proportion of not being in the labor force than those children who were not at risk of developmental delays from birth to 8–9 years (Fig. 2). Fathers of children at risk of one or two developmental delays were consistently more likely to be not in the labor force from birth to 14–15 age (Figure S2). Interestingly, no similar patterns were found for fathers of children at risk of ≥3 developmental delays (Figure S2).

Table 3 displays the adjusted odds ratio of mothers being not in the labor force. The results show that mothers of children at risk of communication, relating, learning, coping, or behaving developmental delays had higher odds of being out of the labor force than mothers of children not at risk of developmental delay at each age across 2–3 to 14–15 years. Similar patterns were found for mothers who had children at risk of there or more developmental delays. Notably, the significance and magnitude of these associations differ. The highest odds were observed for mothers of children at risk of *mobility* delay, when children were aged 4–5 years (aOR: 4.71, 95% CI: 1.31–16.95) and 6–7 years (aOR: 5.33, 95% CI: 1.56–18.22).

**Table 3:** Adjusted odds ratio of *mothers* being not in the labor force by children’s health status at age 4–5 years and age of children

Health status at age 4–5 years	Children’s age (years) at each wave							
	0–1 Wave 1	2–3 Wave 2	4–5 Wave 3	6–7 Wave 4	8–9 Wave 5	10–11 Wave 6	12–13 Wave 7	14–15 Wave 8
Not at risk of developmental delays	REFERENCE	REFERENCE	REFERENCE	REFERENCE	REFERENCE	REFERENCE	REFERENCE	REFERENCE
At risk of *any* developmental delays	0.97 (0.60–1.59) *p* = 0.9132	1.20 (0.80–1.81) *p* = 0.3776	1.65 (1.10–2.47) *p* = 0.0150	1.20 (0.77–1.87) *p* = 0.4331	1.30 (0.83–2.03) *p* = 0.2606	1.36 (0.83–2.22) *p* = 0.2213	1.77 (1.10–2.85) *p* = 0.0196	1.82 (1.09–3.05) *p* = 0.0232
At risk of *one* developmental delay	1.15 (0.49–2.72) *p* = 0.7454	1.15 (0.59–2.23) *p* = 0.6858	1.45 (0.75–2.80) *p* = 0.2646	1.20 (0.61–2.36) *p* = 0.5943	0.88 (0.39–1.96) *p* = 0.7480	1.21 (0.55–2.67) *p* = 0.6312	1.45 (0.65–3.24) *p* = 0.3634	1.07 (0.43–2.70) *p* = 0.8849
At risk of *two* developmental delays	1.42 (0.48–4.19) *p* = 0.5232	1.37 (0.51–3.70) *p* = 0.5363	1.68 (0.63–4.44) *p* = 0.2971	0.70 (0.19– 2.65) *p* = 0.6008	0.84 (0.29–2.40) *p* = 0.7439	1.57 (0.50–4.95) *p* = 0.4419	1.64 (0.62–4.31) *p* = 0.3176	3.18 (1.16–8.76) *p* = 0.0249
At risk of *three or more* developmental delays	0.66 (0.33–1.31) *p* = 0.2357	1.20 (0.68–2.10) *p* = 0.5263	1.86 (1.08–3.24) *p* = 0.0245	1.44 (0.77–2.70) *p* = 0.2519	2.16 (1.19–3.92) *p* = 0.0117	1.43 (0.71–2.89) *p* = 0.3223	2.21 (1.13–4.34) *p* = 0.0204	2.19 (1.07–4.51) *p* = 0.0330
At risk of *communication* delay	0.93 (0.55–1.59) *p* = 0.7946	1.23 (0.80–1.92) *p* = 0.3487	1.59 (1.03–2.46) *p* = 0.0371	1.09 (0.67–1.78) *p* = 0.7211	1.15 (0.71–1.87) *p* = 0.5761	1.32 (0.78–2.23) *p* = 0.3075	1.71 (1.03–2.83) *p* = 0.0394	1.69 (0.96–2.98) *p* = 0.0676
At risk of *mobility* delay	0.54 (0.07–4.21) *p* = 0.5545	1.70 (0.25–11.66) *p* = 0.5886	4.71 (1.31–16.95) *p* = 0.0179	5.33 (1.56–18.22) *p* = 0.0076	2.70 (0.69–10.54) *p* = 0.1526	0.66 (0.06–7.62) *p* = 0.7393	0.91 (0.07–11.58) *p* = 0.9417	1.26 (0.11–14.92) *p* = 0.8544
At risk of *self-care* delay	0.46 (0.17–1.24) *p* = 0.1240	0.99 (0.45–2.16) *p* = 0.9697	1.19 (0.52–2.73) *p* = 0.6879	1.17 (0.50–2.75) *p* = 0.7219	1.78 (0.79–4.01) *p* = 0.1652	0.72 (0.22–2.40) *p* = 0.5967	1.70 (0.64–4.48) *p* = 0.2875	1.29 (0.41–4.10) *p* = 0.6680
At risk of *relating* delay	0.71 (0.35–1.43) *p* = 0.3342	1.41 (0.77–2.60) *p* = 0.2666	1.76 (0.96–3.22) *p* = 0.0683	1.26 (0.60–2.64) *p* = 0.5395	2.11 (1.07–4.16) *p* = 0.0303	1.58 (0.74–3.39) *p* = 0.2381	1.81 (0.83–3.93) *p* = 0.1361	2.34 (1.07–5.11) *p* = 0.0330
At risk of *learning* delay	0.95 (0.43–2.11) *p* = 0.9055	1.45 (0.77–2.75) *p* = 0.2520	2.07 (1.09–3.93) *p* = 0.0262	1.36 (0.65–2.82) *p* = 0.4133	1.55 (0.80–3.03) *p* = 0.1979	1.70 (0.79–3.66) *p* = 0.1741	2.61 (1.36–5.01) *p* = 0.0040	3.09 (1.46–6.53) *p* = 0.0032
At risk of *coping* delay	0.67 (0.33–1.38) *p* = 0.2769	1.23 (0.68–2.21) *p* = 0.4967	1.50 (0.82–2.75) *p* = 0.1848	1.16 (0.54–2.48) *p* = 0.7058	1.56 (0.75–3.26) *p* = 0.2389	1.34 (0.60–2.97) *p* = 0.4782	1.32 (0.59–2.96) *p* = 0.5041	1.32 (0.59–2.96) *p* = 0.5029
At risk of *behaving* delay	0.67 (0.31–1.46) *p* = 0.3153	1.13 (0.60–2.14) *p* = 0.7006	1.73 (0.93–3.22) *p* = 0.0842	1.46 (0.74–2.87) *p* = 0.2787	2.14 (1.11–4.12) *p* = 0.0225	1.11 (0.48–2.60) *p* = 0.8038	2.00 (0.94–4.27) *p* = 0.0714	2.53 (1.21–5.29) *p* = 0.0135

Adjusted for mother’s age, education attainment, and employment status at time of birth, as well as marital status at the current wave

*Shaded cells indicate significance at the 0.05 level

Table 4 shows the adjusted hazard ratio of *returning* to the labor force after leaving for mothers. All mothers of children at risk of developmental delays had a significantly lower hazard ratio of *returning* to the labor force during the follow-up, except mothers of children who were at risk of two developmental delays. The median length of time *returning* to the labor force was two years for mothers whose children were not at risk of developmental delays, whereas those whose children were at risk of developmental delays had double or three times this length of time, except for mothers who had children at risk of *self-care* delay stayed at the same length.

**Table 4:** Adjusted hazard ratio of *returning* to the labor force for *mothers* by children’s health status at age 4–5 years

Health status at age 4–5 years	Median length of time returns to the labor force (years)	Adjusted hazard ratio	95% CI	p-value
Not at risk of developmental delays	2	REFERENCE		
At risk of *any* developmental delays	4	0.69	0.67–0.71	< 0.0001
At risk of *one* developmental delay	4	0.74	0.72–0.77	< 0.0001
At risk of *two* developmental delays	6	1.02	0.97–1.08	0.3942
At risk of *three or more* developmental delays	6	0.51	0.49–0.53	< 0.0001
At risk of *communication* delay	4	0.73	0.71–0.74	< 0.0001
At risk of *mobility* delay	6	0.20	0.16–0.26	< 0.0001
At risk of *self-care* delay	2	0.48	0.45–0.52	< 0.0001
At risk of *relating* delay	6	0.66	0.63–0.70	< 0.0001
At risk of *learning* delay	6	0.64	0.61–0.67	< 0.0001
At risk of *coping* delay	4	0.66	0.63–0.70	< 0.0001
At risk of *behaving* delay	4	0.54	0.51–0.56	< 0.0001

Adjusted for mother’s age, education attainment, and employment status at time of birth, as well as marital status at the current wave

CI: Confidence Interval

## Discussion

Our results show that mothers of children at risk of each type of developmental delay (expect mobility and self-care) at age 4–5 years had greater odds of being out of and not *returning* to the workforce from children aged 2–3 to 14–15 years, compared to mothers of children were not at risk of developmental delay. Paternal labor force participation was slightly influenced, however, the number of fathers out of the labor force was small, so we were unable to conduct multivariate regressions. These findings support other studies which have indicated that the labor force participation for mothers was reduced because of having a child in poor health, whereas fathers were not significantly affected (Breslau et al., 1982; Corcnan et al., 2005; Kuhlthau & Perrin, 2001; Noonan et al., 2005; Powers, 2003; Wondemu et al., 2022). A possible explanation for the difference is mothers still take a higher proportion of responsibility for caring for and raising children (Minister & Cabinet, 2017).

The current study also found differences in parental labor force participation at various time points for different types of developmental delays, suggesting that parents are differentially affected when their children are of certain ages. In general, there was a declining trend of mothers being out of the labor force as the child’s age increased (except mothers of children at risk of mobility delay). However, the fluctuations during the declining trend reveal a complex pattern of parental involvement for children at risk of different developmental delays, where the child may require greater levels of care at different times in their life. For example, our findings showed that mothers leaving the labor force peaked when their children aged 4–5 years (preschool). It may indicate the additional needs of children at risk of developmental delays during the transition from kindergarten to primary school.

Parental participation in the labor force is vital for generating income for families. Being out of the labor force due to caring responsibilities will likely cause financial strain to jobless families, which is of particular concern for families of children with chronic health conditions who require access to often-expensive health services (Ouyang et al., 2014; Rogge & Janssen, 2019; Saunders et al., 2015). This demonstrates how poor health can lead to poverty and further compromise the accessibility to medical services, resulting in a cyclical relationship (Essue et al., 2011; Hynd et al., 2008; Jan et al., 2012; Jeon et al., 2009).

### Policy Implications

In the absence of income from employment, access to government-provided welfare or social security payments (e.g., low income support payments) is essential for supplementing the income of parents who have children at risk of developmental delays. Similarly, government subsidization of additional health services required by the children at risk of developmental delays (e.g., speech therapy, occupational therapy and physiotherapy), and the cost of additional educators or carers, not only support the children to access these services without cost acting as a barrier, but also reduce the financial burden for their parents.

On the other hand, accessible, flexible and affordable quality childcare and kindergarten services also affect parents’, especially mothers’ decision to participate in the labor force. Particularly, the provision of professional development and other assistance (e.g., extra teacher aide time) to support children with additional needs. Thus public spending on these services and policies that reduce relevant costs via tax reductions, cash benefits, or subsidized direct delivery of early childhood education and care, might enhance the parental participation rate (del Carmen Huerta et al., 2011; Szabo-Morvai & Lovasz, 2017). In addition, participating in a quality early childhood education and care program can provide children at risk of developmental delay opportunities to develop and improve their social, communication and play skills from an early age. Flexible job schedules would be of benefit to parents having children at risk of developmental delays.

Currently, several programs and policies (e.g., improving paid parental leave scheme and increasing work flexibility) have been proposed by the Australian Government, aiming to reduce the gap in participation rates between women and men (Minister & Cabinet, 2017), however, little effort has been made targeting parents of children at risk of developmental delays.

### Strengths and Limitations

The strength of our analyses is that they drew on data from a contemporary and high-quality 14–15 years longitudinal dataset of children and parents. Nonetheless, there are a number of limitations. The health status was defined based on parent’s reports rather than clinical diagnosis. Notably, other chronic health conditions that might affect our findings are not included in our analysis due to relevant data were not collected until children aged 10–11 years. For example, neurological conditions often co-occur with developmental delays (Khan & Leventhal, 2020). The exclusion of other potential confounders (e.g., subsequent pregnancies) due to no relevant data being collected may also compromise the robustness of our results. Also, it is possible that a child was at risk of developmental delays at an earlier age (< 4 years) and affected parental labor force participation. However, we were unable to capture it as the exposure data was collected since children aged 4–5 ages (Wave 3).

## Conclusion

In conclusion, this study examined how having a child at risk of a particular developmental delay influences the rate of parental labor force participation. The results of this study illustrate that having a child at risk of developmental delays negatively influences both maternal and paternal labor force participation, with different extents affected by types of developmental delays and children’s age. This study also highlights the need to stratify children’s developmental delays into various types and to have a long follow-up period, with parental labor force participation being influenced differently over time.

### Electronic supplementary material

Below is the link to the electronic supplementary material.

Supplementary Material 1

## Data Availability

Data is available by applying through the Australian Data Archive (ADA). https://dataverse.ada.edu.au/dataverse/ncld.

## Code Availability

The analyses were undertaken using SAS V9.4. The code used for this study is available from the corresponding author on reasonable request.
