# Supplementary material for: Long-Term Effect of Having a Child at Risk of Developmental Delays on Parental Labor Force Participation
Source: Matern Child Health J. 2024 Feb 9;28(6):1052–60. doi: 10.1007/s10995-024-03897-4 (PMC11058796; doi:10.1007/s10995-024-03897-4)
Supplement: Supplementary file 1 — Supplementary Material 1 [file 10995_2024_3897_MOESM1_ESM.docx]

Fig. S1 Proportion of *mothers* not in the labor force by the number of risk of developmental delays at age 4-5 years and age of children

Fig. S2 Proportion of *fathers* not in the labor force by the number of risk of developmental delays at age 4-5 years and age of children
